# Hemangioma of the prostate - an unusual cause of lower urinary tract symptoms: Case report

**DOI:** 10.1186/1471-2490-11-4

**Published:** 2011-04-13

**Authors:** Reza R Serizawa, Nis Nørgaard, Thomas Horn, Henrik Vibits

**Affiliations:** 1 Department of Pathology, Copenhagen University Hospital at Herlev, Herlev Ringvej 75, Herlev, 2730 Denmark; 2 Department of Urology, Copenhagen University Hospital at Herlev, Herlev Ringvej 75, Herlev, 2730, Denmark; 3 Department of Pathology, Bispebjerg Hospital, Bispebjerg Bakke 23, Copenhagen NV, 2400, Denmark

**Keywords:** Hemangioma, Prostate, Transrectal ultrasound, Transurethral Resection of Prostate, Urinary bladder neck obstruction

## Abstract

**Background:**

Hemangioma of the prostate gland is extremely rare and only a few cases have been reported. There have been several cases of hemangioma of posterior urethra, urinary bladder and periprostatic plexus in the literature, all presenting with hematuria or hematospermia. Diagnosis of prostatic hemangioma is difficult due to its rarity and unspecific symptoms such as hematuria, hematospermia or lower urinary tract symptoms. It cannot be detected by conventional examinations such as cystoscopy or standard rectal ultrasonography.

**Case presentation:**

We present a case of prostatic hemangioma in an 84-year old male presenting with lower urinary tract symptoms. Bleeding has not been a feature in our case and diagnosis was not made until after operation. The patient was treated as a case of bladder neck outflow obstruction with transurethral resection of prostate gland and simultaneous bladder neck incisions. A period of self-catheterization was instituted due to postoperative urinary retention as the result of detrusor insufficiency.

**Conclusion:**

Hemangioma of prostate gland is extremely rare and symptomatic prostatic hemangioma should be treated either by transurethral resection of prostate or laser evaporation.

## Background

Hemangioma of the bladder or posterior urethra presenting with hematuria, hematospermia or urethral bleeding have been sporadically reported [1,2]. Hemangioma arising in prostatic tissue causing lower urinary tract symptoms (LUTS) without hematuria or hematospermia is extremely rare [3,4]. The diagnosis of prostatic hemangioma is very difficult and in the reported cases, diagnosis has been made postoperatively on the basis of histopathology. Diagnosis of prostatic hemangioma is not possible by conventional radiographic techniques such as standard rectal ultrasonography of prostate gland. More sophisticated techniques such as superselective angiography of inferior vesical artery or Doppler ultrasonography are needed to establish the diagnosis [2,5]. Preoperative diagnosis is of great advantage in planning the right treatment and avoiding complications. Prostatic hemangioma may be included in the list of differential diagnoses, in patients having LUTS associated with unexplained hematuria, hematospermia or urethral bleeding. We describe a case of prostatic hemangioma in an 84 years old patient presenting with LUTS without macroscopic hematuria, hematospermia or urethral bleeding.

## Case presentation

An 84 year old male was referred with LUTS, complaining of frequency of micturation, nocturia (2-3 times) and weak stream. The patient scored 30 points on Danish Prostatic Symptom Score (DAN-PSS) and had been treated with alfa blockers by the General practitioner without any symptomatic effect. Rectal examination revealed a normal sized non tender prostate with a normal consistency. Urethral secretion showed traces of blood. Serum prostate specific antigen level was 1.6 ng/ml (normal 0-4 ng/ml). Pressure flow studies showed infravesical outflow obstruction (maximum flow rate (Qmax): 5.4 ml/second and post void residual urine volume (PVR): 109 ml) and transrectal ultrasound revealed a 34 ml prostate. On the basis of symptoms and pressure-flow studies it was decided to perform a transurethral resection of prostate.

**Figure 1:**
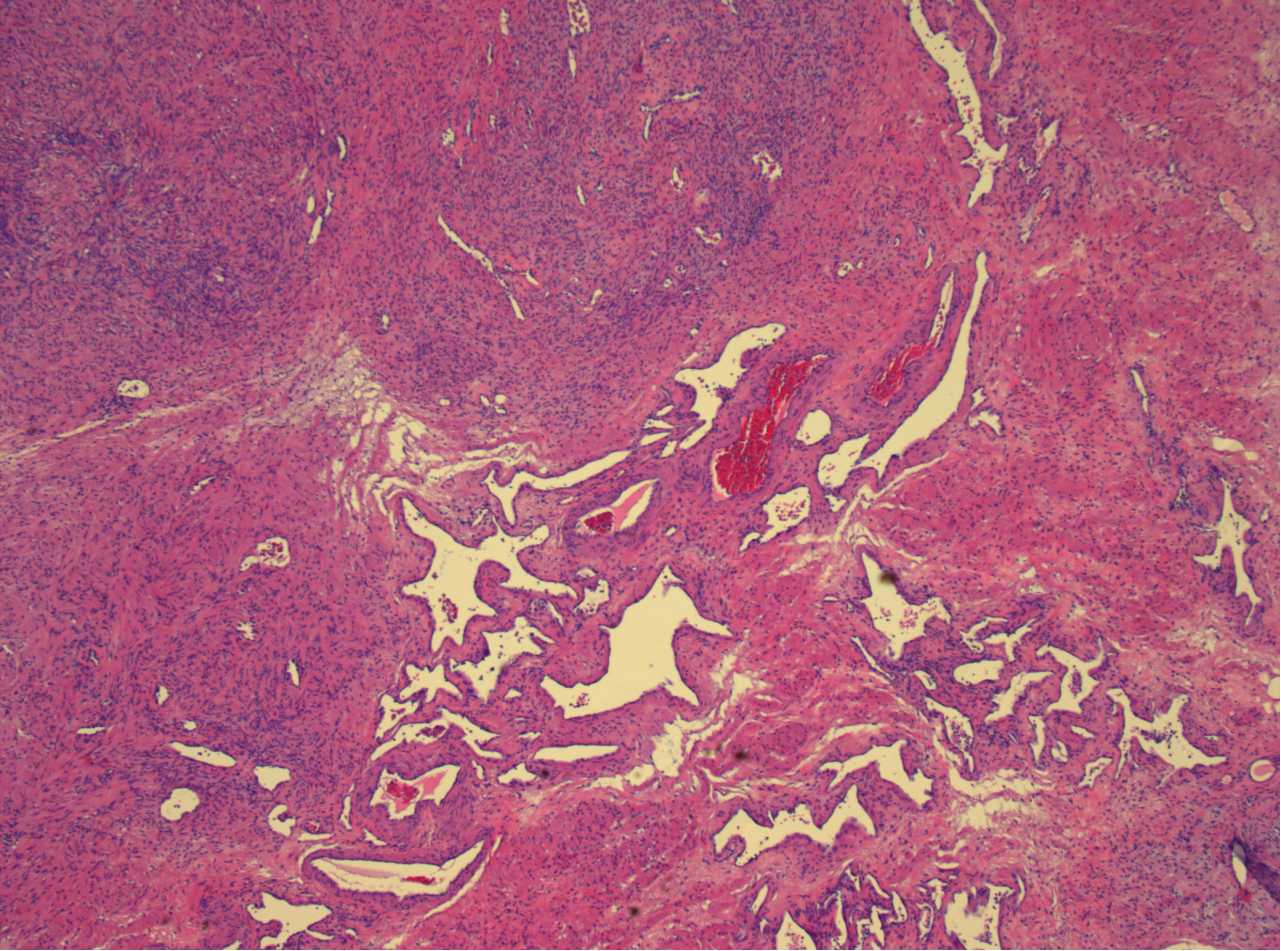
**Section of prostate with a cellular myofibrous stroma containing irregular, thin-and thick walled vessels containing blood**.

**Figure 2:**
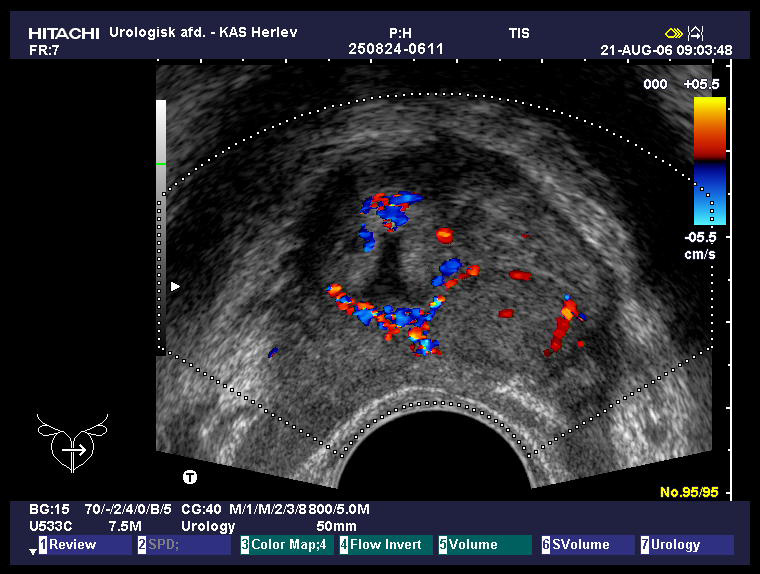
**Colour Doppler transrectal ultrasound, transverse image of the prostate**. Surrounding the TURP resection cavity tiny blood vessels representing the unresected part of the haemangioma are seen.

Cystoscopy showed normal collicular distance and a contracted bladder neck. Bladder neck was incised at the 5 and 7 o'clock positions and transurethral resection of the prostate (around 5 grams) was carried out. There was no undue bleeding per- or postoperatively. Pathology showed a hemangioma of prostate gland (Figure 1) occupying around 30% of the resected material. Postoperative high resolution transrectal ultrasound showed a resection cavity and unresected hemangioma with a total volume estimated at 25 ml (Figure 2). Consequently the obstruction was probably caused by the hemangioma. At follow-up 7 days after surgery the patient complained of a slow stream and difficulty initiating micturation. Postvoid residual urine was recorded at 1700 ml. The patient was treated with indwelling catheter for 14 days and subsequently performed intermittent self-catheterization for six months in order to monitor residual urine, though after two months the patient only needed to self-catheterize once or twice a week. At the final control six months after TURP the patient was symptom free with a Qmax of 17 ml/second and a residual urine volume of 50 ml. We would have offered a repeat TURP if the patient had a symptomatic infravesical obstruction at the final check.

## Discussion

Hemangioma of the urinary tract is uncommon with the majority of cases located in the kidney, ureter or bladder [1]. A number of cases of hemangioma occurring in prostatic urethra and presenting with hematuria, hematospermia or postejaculation urethral bleeding have also been reported [2]. Hemangioma located exclusively in the prostate is extremely rare and only a few cases have previously been reported [3,4]. Sundarasivarao et al. reported a case where the patient presented with chronic urine retention and the diagnosis of prostatic hemangioma was only made after prostatic enucleation [3]. Rivoir and Kaslaris reported a case of prostatic hemangioma in a 65-year-old patient with five years history of nocturia and a single episode of temporary retention of urine. Cystoscopy showed bladder trabeculation and severely injected bladder mucosa. The urine contained a large number of erythrocytes. The patient was subjected to perineal intracapsular prostatectomy, which was complicated by intraoperative uncontrollable bleeding which despite packing and use of spongostan resulted in the patient's death the day after operation. Histology showed capillary hemangioma [4]. Whereas diagnosis of prostatic hemangiona is relatively straightforward for the pathologist, it poses a diagnostic and therapeutic challenge to the urologist. Prostatic hemangioma has no specific clinical features apart from hematuria and heamtospermia, which can occur in many other conditions. If prostatic hemangioma presents as LUTS, as it has done in the present case, the only feature that distinguishes it from benign prostate hyperplasia is association of unexplained hematuria or hematospermia. Prostatic hemangioma can be suspected at cystoscopy by visualization of bluish red areas in the bladder or prostatic urethra [2,5]. A transrectal power Doppler ultrasonography is a useful tool in diagnosing prostatic hemangioma in patients with unexplained hematospermia and/or hematuria, but we do not consider it useful in routine clinical practice unless cystoscopy suggests the presence of a hemangioma [2]. Perhaps Doppler ultrasonography using contrast could be even more helpful showing the typical wash in wash out pattern seen in hemangiomas in the liver, but that remains to be examined. Routine rectal ultrasound used to measure prostatic volume did not reveal the presence of a hemangioma in our patient. A definitive preopertative diagnosis can usually be achieved through arteriography of the internal iliac arteries [6]. Treatment of hemangioma in the bladder and prostate has varied from electrocautery, Nd-YAG laser to radiation or in cases of periprostatic hemangioma with selective arterial embolization [5,7,8]. In our case hemangioma was located in the prostatic tissue. The symptoms and the urodynamic investigations were similar to those of infravesical outflow obstruction. Cystoscopy did not reveal any discoloration in the prostatic urethra or on prostatic lobes. The patient was treated as a case of prostatic hyperplasia with transurethral resection. The patient developed postoperative retention of urine and this was thought to be due to detrusor insufficiency and therefore a period of conservative treatment with self-cathererization was adopted. Prostatic hemangioma presenting as LUTS can be treated as an ordinary symptomatic prostatic enlargement by transurethral resection or other modalities such as laser evaporation. Symptomatic prostatic hemangioma if diagnosed preoperatively should be treated with TURP. We have no evidence that laser or bipolar evaporation are superior to a classic TURP. Nevertheless one might choose to use laser or bipolar techniques if possible. If a prostataic hemangioma is discovered during a TURP we recommend one should carry on with a careful transurethral resection of prostate, alternatively one could convert the procedure to laser or bipolar technique if possible. One should be cautious in performing open prostatectomy in the case of symptomatic prostatic hemangioma because of risk of undue bleeding [4]. To our knowledge hemangioma's never become malignant, but after partial removal some regeneration with growth may occur.

## Conclusion

Hemangioma of prostate gland as a cause of lower urinary tract symptoms is extremely rare and there have been to our knowledge only two cases reported in the literature. High index of suspicion is required preoperatively in diagnosis of this condition, as adoption of wrong operative treatment may lead to fatal complications. We have in our discussion pointed out the clues which may help to obtain the right diagnosis.

## Consent

Written informed consent was obtained from the patient for publication of this case report and any accompanying images. A copy of the written consent is available for review by the Editor-in-Chief of this journal.

## Competing interests

The authors declare that they have no competing interests.

## Authors' contributions

Each author has contributed with 25% to the paper. RS viewed the pathological specimen with TH and drafted the manuscript. NN carried out the ultrasongraphy and contributed to the manuscript. HV performed the operation and clinical follow-up and contributed to the manuscript. TH participated in the draft of the manuscript and viewed the pathological specimen with RS. All authors read and approved the final manuscript.

## Pre-publication history

The pre-publication history for this paper can be accessed here:

http://www.biomedcentral.com/1471-2490/11/4/prepub
